# 

**Published:** 1921-02-12

**Authors:** 


### THE HOSPITAL

**The Modern Newspaper of Administrative Medicine and Institutional Life.**

Telegraphic Address: OSPEDALE, RAND, LONDON. Telephone Number: 2734 GERRARD.

No. 1810, Vol. LXIX. | Saturday, February 12th, 1921. | PRICE TWOPENCE.

